# Associations between cigarette smoking status and health-related physical fitness performance in male Taiwanese adults

**DOI:** 10.3389/fpubh.2022.880572

**Published:** 2022-08-18

**Authors:** Chien-Chang Ho, Po-Fu Lee, Shu Xu, Chang-Tsen Hung, Yan-Jhu Su, Chi-Fang Lin, Min-Chen Wu, Yun-Tsung Chen

**Affiliations:** ^1^Department of Physical Education, Fu Jen Catholic University, New Taipei City, Taiwan; ^2^Research and Development Center for Physical Education, Health, and Information Technology, College of Education, Fu Jen Catholic University, New Taipei City, Taiwan; ^3^Sports Medicine Center, Fu Jen Catholic Hospital, New Taipei City, Taiwan; ^4^Department of Leisure Industry and Health Promotion, National Ilan University, Yilan County, Taiwan; ^5^Department of Gerontology, University of Massachusetts Boston, Boston, MA, United States; ^6^Department of Health and Leisure Management, Yuanpei University of Medical Technology, Hsinchu, Taiwan; ^7^Department of Physical Education and Sport Sciences, National Taiwan Normal University, Taipei City, Taiwan; ^8^Office of Physical Education, Chung Yuan Christian University, Taoyuan, Taiwan

**Keywords:** physical fitness, cigarette smoking, adults, Taiwan, cross-sectional study

## Abstract

**Background:**

The highest proportion of smoking behavior occurs in male adults in Taiwan. However, to our knowledge, no study has investigated the relationship between smoking behavior and health-related physical fitness according to education level, health status, betel nut-chewing status and obesity in male adults aged 18 years or older in Taiwan.

**Aims:**

This study aimed to determine the associations between cigarette smoking and health-related physical fitness performance in male Taiwanese adults.

**Methods:**

This was a cross-sectional study conducted on 27,908 male adults (aged 23–64 years) who participated in Taiwan's National Physical Fitness Survey 2014–2015. Data from a standardized structured questionnaire, anthropometric variables, and health-related physical fitness measurements were analyzed. Individuals were categorized as never smoking cigarettes, former smoker, and current smoker. Multiple linear regression analysis was performed to evaluate the association between cigarette smoking and health-related physical fitness performance.

**Results:**

Never smoking group exhibited a lower (*p* < 0.05) proportion of abdominal obesity, higher (*p* < 0.05) proportion of perceived good health status, and greater (*p* < 0.05) performance in 1-min sit-up and sit-and-reach tests when compared with current smoking and former smoking group. Former smoking group had the highest (*p* < 0.05) performance in 3-min step test among all groups. Current smoker was significantly negatively (*p* < 0.05) associated with 3-min step, 1-min sit-up and sit-and-reach tests. Notably, former smoker was significantly positively (*p* < 0.05) associated with 3-min step and 1-min sit-up tests, but still negatively (*p* < 0.05) associated with sit-and-reach performance.

**Conclusion:**

Current smoker was associated with an increased the risk of abdominal obesity, reduced the perceived health status and health-related physical fitness performance. Quitting smoking had beneficial effect on the perceived good health status, cardiorespiratory and muscular fitness in male Taiwanese adults, but not on flexibility performance. Further research on the ameliorate mechanism underlying this phenomenon is warranted.

## Introduction

Health-related physical fitness, including body composition, cardiorespiratory fitness, muscular strength and endurance as well as flexibility, influences different health aspects, such as susceptibility to hypertension, insulin resistance, cardiovascular diseases, osteoporosis and cancer (1, 2). Higher levels of cardiorespiratory fitness and upper- and lower-body muscular strength are associated with a lower risk of all-cause mortality in healthy adults (3–5). Furthermore, greater flexibility may increase the joint range of motion and reduce the risk of metabolic syndrome (MS) (6, 7).

Maximal oxygen uptake (VO_2_max) is the standard measure of cardiorespiratory fitness and can be obtained from measurements during maximal and submaximal exercise testing, such as treadmill, field and step tests (1). Compared with the maximal exercise test, the 3-min step test is an inexpensive, time-efficient and valid method for predicting cardiopulmonary function in a laboratory setting (8). In addition, the 1-min sit-up test predicts trunk muscular endurance (9) and is negatively associated with fasting blood glucose levels and MS risk (10–12). Additionally, the sit-and-reach test is a widely accepted measure of hamstring flexibility, which reduces lower back pain and muscle strain and prevents falls (13). Notably, smoking and second-hand smoke were associated with cancer (e.g., oral cavity, lung, liver, and kidney cancers), stroke, diabetes, cardiovascular diseases and chronic obstructive pulmonary disease (14). Relatively limited information is available on the relationship between smoking status and health-related physical fitness (15).

Tobacco use (9%) is the second greatest global risk factor for mortality; other risk factors include high blood pressure (13%), high blood glucose (6%) and physical inactivity (6%) (16). The greatest reduction in life expectancy between ages 40 and 85 years is attributed to smoking (4.8 years), followed by diabetes (3.9 years), physical inactivity (2.4 years) and hypertension (1.6 years). In addition, smoking is responsible for more than 8 million annual deaths worldwide, and more than 1.2 million annual deaths are the result of exposure to second-hand smoke (17). However, most smokers do not understand the health risks of smoking, and without cessation support, only 4% will successfully quit (17). Therefore, more research is warranted to examine whether quitting tobacco use improves health-related physical fitness, thereby improving health aspects and reducing the risk of disease and death.

Recent study indicated that tobacco smoking was significantly negatively associated with 3-km running and 2-min push-up among military young adults after adjusting for confounding factors (age, body mass index, heart rate, service specialty, hypertension status, and exercise frequency) (18). A study shown that the use of properly adjusted confounding factors (e.g., gender, health status, occupation, and education) is necessary to avoid overestimate or underestimate the real magnitude of an association, thereby preventing bias and distortion (19). In addition, quitting smoking increased the repetitions of 20-m shuttle run, but it had no beneficial effects on 50-m sprint, grip strength, 1-min sit-up and sit-and-reach performance in Korean young adults (20).

In Taiwan, the prevalence of tobacco smoking in 2021 was 23.1% and 2.9% for men and women, respectively (21). Moreover, the highest proportion (39.7%) of smoking behavior was observed among men between the ages of 46 and 50 years (21). However, to our knowledge, no study has investigated the relationship between smoking status and health-related physical fitness according to education level, health status, betel nut-chewing status and obesity in male adults aged 18 years or older in Taiwan.

Therefore, the purposes of the present study were: (1) to investigate the association between smoking status (never-, current- and former-smoking) and health-related physical fitness and identify factors that influence this relationship among male adults in Taiwan; (2) to examine whether there is a beneficial effect on health-related physical fitness after quitting smoking. We hypothesized that current smoking would result in a lower health-related physical fitness than those never- and former-smoker.

## Materials and methods

### Study design and data sources

The data used for this cross-sectional study were obtained from Taiwan's National Physical Fitness Survey (TNPFS) conducted by the Sports Administration, Ministry of Education in Taiwan. The validated protocol and tool of this survey have been published elsewhere (21–26). Here, a brief summary of the survey methods is presented. All participants in this survey were recruited through age- and sex-stratified convenience sampling from 46 physical fitness test stations in 22 cities and counties in Taiwan between October 2014 and March 2015. This survey included a face-to-face interview followed by a standardized structural questionnaire, anthropometric measurements, and health-related physical fitness tests conducted by trained examiners and medical specialists (usually nurses or doctors). These data collected in TNPFS were deidentified secondary data, which were administrated by the Sports Cloud: Information and Application Research Center of Sports for All, Sport Administration, Ministry of Education in Taiwan and released to the public for research purposes. Detailed information about TNPFS is available at https://isports.sa.gov.tw/index.aspx. This study was conducted in accordance with the Declaration of Helsinki, and the protocol was approved by the Institutional Review Board of Fu Jen Catholic University in Taiwan (FJU-IRB C110113).

### Eligibility criteria for study participants

Before the TNPFS data collection, the ineligible participants have excluded according to the following criteria: (1) systolic blood pressure ≥140 mmHg or diastolic blood pressure ≥90 mmHg; and (2) currently/previously have heart disease, hypertension, chest pain, vertigo or musculoskeletal disorders. Thus, the TNPFS database 2014–15 includes 62,586 adults aged 23–64. Furthermore, in order to select the eligible participants for the present study, female and non-Taiwanese participants were excluded. In addition, data with missing values were also excluded from the further analysis. Finally, 27,908 male Taiwanese adults aged 23–64 left for the present study.

### Data collection

For the present analysis, data on sociodemographic characteristics (age, sex, education, monthly income, and marital status), cigarette smoking status, betel-nut chewing habits, perceived health, and anthropometric variables were recorded by well-trained examiners using face-to-face interviewing.” to indicate that the trained examiners filled the data in face-to-face interviews. Education was divided into three categories: elementary school or lower, junior or senior high school, and college or higher. Monthly income was divided into three categories: ≤20,000 NTD, 20,001–40,000 NTD, and ≥40,001 NTD. Marital status was divided into three categories: married, never married, and divorced/separation/widowed. Betel-nut chewing habits were characterized in one of the following categories: “never users” (those who never established the habit), “former users” (those who had the habit but had since quit), and “current users” (those who continued with the habit). Self-reported health status was divided into three categories: excellent or good, fair, and very bad or poor. After participants completed the interview questionnaire, anthropometric variables were measured, including body weight, height, waist circumference (WC), and hip circumference (HC). Body weight was measured in light underclothes to an accuracy of 0.1 kg using a scale. Height was measured to an accuracy of 0.1 cm using a wall-mounted metal measuring tape and an acute-angled head piece while the participants stood against a plumb-checked vertical wall with their shoes removed. Then, BMI was calculated as body weight (kg) divided by the square of the height (m^2^). Obesity was classified according to the cutoff BMI classification for Taiwanese adults, which was adopted as suggested by the Health Promotion Administration, Ministry of Health and Welfare in Taiwan: individuals were identified as underweight (BMI < 18.5 kg/m^2^), normal weight (18.5 ≤ BMI < 24 kg/m^2^), overweight (24 ≤ BMI < 27 kg/m^2^) or obese (BMI ≥ 27 kg/m^2^) (27). The WC measurements (measured to the nearest 0.1 cm) were performed twice halfway between the lowest rib and iliac crest after a normal exhale, and the mean value was used. The HC measurements (measured to the nearest 0.1 cm) were performed twice at the site of the largest convexity of the buttocks below the hip plates, and the mean value was used. Then, WHR was calculated as WC (cm) divided by HC (cm).

### Cigarette smoking status

Cigarette smoking status was obtained *via* a self-report questionnaire at the interview. Participants were categorized as never smokers if they smoked < 1 g of tobacco (i.e., 1 cigarette) per day, current smokers (> 1 g of tobacco per day) (28), and former smokers (i.e., those who had previously smoked but successfully quit for more than 1 year).

### Health-related physical fitness measures

After the interview, questionnaire and anthropometric measurements, health-related physical fitness measurements were tested in the following order with a sufficient rest period (3–5 min) between tests: muscle strength and endurance by the 1-min sit-up test (reps/min) (29), flexibility by the sit-and-reach test (cm) (30), and cardiorespiratory endurance by the 3-min step test (31). All the tests were performed only once, except the sit-and-reach test, which was performed twice; the higher of the two measurements was recorded. In order to achieve the validity and reliability of the outcome measures, these health-related physical fitness tests were conducted by well-trained examiners who attended a regional training seminar and passed a certification test on standardized procedures, as reported in previous studies (21–26). To achieve optimal health-related physical fitness performance, each participant was asked to avoid any other vigorous physical activity or exercise training before the tests and was allotted a 10 min warmup period (32, 33).

### Statistical analysis

Statistical analyses were performed using SAS version 9.4 (SAS Institute., Cary, NC, USA) software. Data analysis was performed on 27,908 male participants which met the sample sampling requirement. Descriptive analyses included means ± standard deviations (SD) for continuous variables and percentages for categorical variables. The normal distribution of data was examined by using the Shapiro–Wilk test. Demographic characteristics and health-related physical fitness measurements were analyzed for Chi-square tests and one-way analysis of variance (ANOVA) among the cigarette smoking status groups. When a significant *F* value was found (*p* < 0.05), Tukey's *post hoc* test was performed to determine the differences between the pairs of means. Significant differences between cigarette smoking status groups are considered the potential confounders for the linear regression model adjustment. Multiple linear regression analysis was used to examine the association between cigarette smoking status and health-related physical fitness performance after adjusting for potential confounders such as age, general and abdominal obesity, educational levels, monthly income levels, self-reported health status, and betel nut-chewing habits. All statistical tests were two-tailed and considered statistically significant at *p* < 0.05.

## Results

A total of 27,908 male adults were included in this study. Demographic characteristics and anthropometric indices are presented in Table 1. Many participants (70%) reported that they never smoked. However, 10% of participants of all age groups smoked, and the 45–54-year-old group had the highest proportion of smokers. All participants were divided into groups according to their cigarette smoking status: never, former and current smokers. Significant differences (*p* < 0.05) were shown between groups (never, former, current) on all relevant variables, including age, body weight, height, BMI, obesity status, WC, HC, WHR, abdominal obesity, education level, income level, self-reported health status and betel nut-chewing habits. Of the cigarette smoking status groups, former smokers had the highest BMI, WC, and HC. There was no significant difference between former and current smokers in terms of body weight.

**Table 1:** Characteristics of the study participants according to cigarette smoking status among male Taiwanese adults.

**Variable**	**Cigarette smoking status**			***p***	**Tukey's *post-hoc* test**
	**Never**	**Former**	**Current**		
	**(*n* = 19,674)**	**(*n* = 2,766)**	**(*n* = 5,468)**		
Age (%)				<0.001*	
≥ 24 years	9.00	2.46	8.12		
25–34 years	30.61	16.02	27.67		
35–44 years	26.20	30.08	31.82		
45–54 years	19.00	31.56	21.89		
55–64 years	15.19	19.88	10.50		
Body weight (kg)	71.77 ± 10.47	73.30 ± 9.94	73.21 ± 10.84	<0.001*	F, C > N
Height (cm)	170.67 ± 6.45	170.32 ± 6.08	171.01 ± 6.07	<0.001*	C > N > F
BMI (kg/m^2^)	24.62 ± 3.22	25.26 ± 3.13	25.02 ± 3.39	<0.001*	F > C > N
Obesity status (%)				<0.001*	
Obese	42.42	34.63	37.56		
Overweight	33.89	37.42	33.52		
Normal weight	21.84	27.15	27.07		
Underweight	1.85	0.80	1.85		
WC (cm)	83.98 ± 8.64	86.35 ± 8.34	85.47 ± 8.96	<0.001*	F > C > N
HC (cm)	96.85 ± 6.26	97.35 ± 8.34	97.02 ± 6.42	<0.001*	F > N
WHR	0.87 ± 0.06	0.89 ± 0.06	0.88 ± 0.06	<0.001*	F > C > N
Abdominal obesity (%)				<0.001*	
Yes	25.82	34.16	33.16		
No	74.18	65.84	66.84		
Education level (%)				<0.001*	
Elementary school or lower	1.68	1.54	1.97		
Junior or senior school	19.31	32.16	37.56		
College or higher	79.01	66.30	60.47		
Income level (%)				<0.001*	
≤ 20,000 NTD	16.21	11.18	13.43		
20,001–40,000 NTD	33.69	33.38	39.30		
≥40,001 NTD	50.10	55.44	47.27		
Self-reported health status (%)				<0.001*	
Excellent or good	64.10	60.16	56.21		
Fair	30.07	32.97	36.08		
Very bad or poor	5.83	6.87	7.71		
Betel nut-chewing habits (%)				<0.001*	
Never	99.01	66.43	72.41		
Current	0.65	1.78	14.53		
Former	0.34	31.79	13.06		

BMI, body mass index; C, current smoker; F, former smoker; HC, hip circumference; N, never smoked; NTD, New Taiwan Dollar; SD, standard deviation; WC, waist circumference; WHR, waist-to-hip ratio.

Values expressed as means ± SD for continuous variables and percentage (%) for categorical variables.

* p < 0.05.

Table 2 presents intergroup differences on each health-related physical fitness measurement. All the cigarette smoking status groups showed significant differences on all health-related physical fitness measurements. Those who never smoked received the highest grade on the 1-min sit-up test and sit-and-reach test. In the 3-min step test, former smokers received the highest grade.

**Table 2:** Health-related physical fitness measurements according to cigarette smoking status among male Taiwanese adults.

**Variable**	**Cigarette smoking status**			***p***	**Tukey's *post-hoc* test**
	**Never**	**Former**	**Current**		
	**(*****n*** = **19,674)**	**(*****n*** = **2,766)**	**(*****n*** = **5,468)**		
3-min step test	57.77 ± 10.51	59.08 ± 11.07	56.48 ± 9.95	<0.001*	F > N > C
1-min sit-up test (reps/min)	29.14 ± 10.39	26.96 ± 9.70	28.08 ± 9.71	<0.001*	N > C > F
Sit-and-reach test (cm)	21.75 ± 10.70	20.84 ± 10.75	21.36 ± 10.33	<0.001*	N > F, C

BMI, body mass index; C, current smoker; F, former smoker; N, never smoked; SD, standard deviation; WHR, waist-to-hip ratio.

Values are expressed as the means ± SD.

* p < 0.05.

Table 3 presents the results of multiple regressions on the associations between cigarette smoking status and health-related physical fitness measurements after adjusting for potential confounders. Participants were divided into 3 groups according to their cigarette smoking status. Those who never smoked served as a reference in all regressions (OR = 1.00). After adjusting for potential confounders, a history of smoking (former) had a positive effect on participants' grades on the 3-min step test (β = 1.31, *p* < 0.001) and 1-min sit-up test (β = 0.5, *p* = 0.019). Conversely, current smoking had a negative effect on the grades of the 3-min step test (β = −1.02, *p* < 0.001) and 1-min sit-up test (β = −0.33, *p* = 0.009) compared with those of the reference (never smoked). Smoking (former and current) had a negative effect on the grade of the sit-and-reach test (β = −0.53, *p* = 0.027 and β = −0.37, *p* = 0.036, respectively).

**Table 3:** Multiple regressions for the association between cigarette smoking status and health-related physical fitness after adjusting for potential confounders.

**Variable**	**Cigarette smoking status**	**Model 1 (Age-adjusted)**			**Model 2 (Multivariate-adjusted** ^**a**^ **)**		
		**β**	**SE**	***p***	**β**	**SE**	***p***
3-min step test	Current	−1.29	0.16	<0.001*	−1.02	0.17	<0.001*
	Former	1.00	0.21	<0.001*	1.31	0.23	<0.001*
	Never	Ref.	—	—	Ref.	—	—
1-min sit-up test (reps/min)	Current	−1.14	0.13	<0.001*	−0.33	0.14	0.019*
	Former	0.04	0.18	0.828	0.50	0.19	0.009*
	Never	Ref.	—	—	Ref.	—	—
Sit-and-reach test (cm)	Current	−0.40	0.16	0.014*	−0.37	0.18	0.036*
	Former	−0.49	0.22	0.023*	−0.53	0.24	0.027*
	Never	Ref.	—	—	Ref.	—	—

BMI, body mass index; β, regression coefficient; SE, standard error; WHR, waist-to-hip ratio.

* p < 0.05.

a Adjusted for age, general and abdominal obesity, educational level, monthly income level, self-reported health status, and betel nut-chewing habits.

## Discussion

In this study, we analyzed the relationship between cigarette smoking status and health-related physical fitness performance using data from 27,908 Taiwanese men. The main findings of this study were as follows: (1) current smoking was negatively associated with cardiorespiratory fitness, muscular endurance and flexibility performance; (2) current smoking was resulting in lower health-related physical fitness than those never- and former-smoker; and (3) former smoking was positively associated with cardiorespiratory fitness and muscular endurance but negatively associated with flexibility performance. These findings suggest that quitting tobacco use may restore cardiopulmonary function and muscular fitness in male adults, whereas it had no beneficial effects on hamstring flexibility.

The World Health Organization (WHO) reported that approximately 38% of men aged 15 years and older worldwide were current tobacco users in 2020, and the highest prevalence of tobacco use occurred at ages between 45 and 54 years (34). In this study, 70% of male adults never smoked; the proportion who never smoked was higher for those with college or higher education level (79%), self-reported excellent or good health status (64%), and lower for those with abdominal obesity (25%). Conversely, 20% of male adults smoked, and the highest proportion (32%) of smoking was observed in participants between 35 and 44 years old. Our results suggested that current smoker may increase the risk of abdominal obesity and reduce the perceived good health status when compared never smoker.

Su et al. indicated that smoking was detrimental to 3-km running and 2-min push-up in military adults (18). Kumar et al. reported that nonsmoker athletes had higher flexibility than smokers (35). In contrast, previous studies found no differences in flexibility between smoking and nonsmoking men (20, 36). In the present study, current smoker exhibited a lower performance in 3-min step, 1-min sit-up and sit-and-reach tests when compared with never smoker. A similar study reported that smoking increased ventilatory equivalent for O_2_ (VE/VO_2_) and reduced O_2_ pulse values during submaximal exercise, which may result in reduced pulmonary and muscle tissue gas exchange efficiency and decreased O_2_ carrying capacity, thereby reducing cardiopulmonary fitness performance (37). In addition, the possible mechanisms for the reduction in muscular fitness may be that smoking induces oxidative stress and impairs oxygen delivery to the mitochondria, which may reduce protein synthesis and attenuate the ability of mitochondria to generate ATP, thereby decreasing muscle mass, muscle force and endurance performance (38).

Notably, a previous study suggested that abstinence from smoking may increase VO_2_max performance in males (28). Jeon et al. (20) shown that quitting smoking were reverse the effects of smoking on the repetitions of 20-m shuttle run, but not on 50-m sprint, grip strength, 1-min sit-up and sit-and-reach tests in young adults. In the present study, we found that former smoker had the highest performance in 3-min step test among all groups. However, no beneficial effects were observed on 1-min sit-up and sit-and-reach tests in former smoker. Therefore, we suggested that smoking decreased health-related physical fitness performance, and quitting smoking may improve in cardiopulmonary fitness but not in speed, muscle strength, muscle endurance or flexibility performance in male adults. Further research is required to clarify the mechanisms by which smoking reduces flexibility and whether there is a threshold of abstinence from smoking for recovering health-related physical fitness performance.

A study indicated that smoking was associated with lower weekly exercise frequency and duration than never smoking in both sexes (29). In addition, smoking is negatively associated with cardiorespiratory fitness (including VO_2_max, 3000-meter and 1.5-mile running performance) in male and female adults (18, 39, 40). Similar studies have indicated that current smoking is inversely correlated with repetitions of 2-min sit-ups, curl-ups and push-ups in military adults (18, 39, 41). Kok et al. (42) reported that smoking was inversely related to isokinetic knee extensor and flexor muscle strength performance in healthy adults. In the present study, we observed that current smoking was negatively associated with 3-min step, 1-min sit-up and sit-and-reach tests performance in male adults. Moreover, former smoking was positively associated with 3-min step and 1-min sit-up tests, but still negatively associated with sit-and-reach test performance. Our results suggest that the effects smoking on cardiorespiratory fitness and muscular endurance performance were reversable in former smokers after adjusting for confounding factors (age, sex, self-reported health status, education and betel nut-chewing habits). However, the adverse effects of smoking on hamstring flexibility were not reversible in male Taiwanese adults.

The present study has some limitations. First, this study did not measure the total grams of tobacco smoked per day, Fagerström test for nicotine dependence, or carboxyhemoglobin of the participants. Without these measurements, we could not classify the current smokers into light, moderate or heavy smokers or further analyze the dose–response relationship between tobacco use and health-related physical fitness. Second, our questionnaire did not include the comorbidities (e.g., asthma, heart diseases, diabetes and stroke) (43); thus we cannot compare comorbidity status among three groups, and examine the association between smoking status and comorbidity. Third, we did not ask the reason for quitting tobacco use; therefore, the motivation behind quitting smoking in Taiwanese men remains unclear. Finally, due to the cross-sectional nature of this study, no temporal relationships between cause and effect could be evaluated. Future studies should utilize a longitudinal study design to elucidate the clinical importance of strength and conditioning training in increasing health-related physical fitness and further reversing the effects of smoking.

## Conclusion

In summary, this study demonstrated that current smoking was associated with decreased cardiopulmonary function, muscular endurance and flexibility performance. Notably, cessation of tobacco use may ameliorate the effects of smoking on cardiorespiratory and muscular fitness in male adults. Therefore, our results call for prevent smoking behavior and urge government to include smoking cessation and treatment services as tobacco control strategies for young adults to improve physical fitness and life quality. Further research on the relevant mechanism for health-related physical fitness improvement following quitting smoking is warranted.

## Data availability statement

The original contributions presented in the study are included in the article/supplementary material, further inquiries can be directed to the corresponding author.

## Ethics statement

Written informed consent was obtained from the individual(s) for the publication of any potentially identifiable images or data included in this article.

## Author contributions

C-CH and Y-TC participated in the design, conducted the statistical analyses, interpreted the data, and drafted the manuscript. P-FL supervised the study, assisted in data interpretation, and critically reviewed the manuscript. SX and C-TH helped in conducting the study and revising the manuscript. Y-JS, C-FL, and M-CW helped to manage and analyze the data. All authors read and approved the final manuscript.

## Funding

This study was supported by a grant from the Ministry of Science and Technology (MOST 109-2410-H-030-059).

## Conflict of interest

The authors declare that the research was conducted in the absence of any commercial or financial relationships that could be construed as a potential conflict of interest.

## Publisher's note

All claims expressed in this article are solely those of the authors and do not necessarily represent those of their affiliated organizations, or those of the publisher, the editors and the reviewers. Any product that may be evaluated in this article, or claim that may be made by its manufacturer, is not guaranteed or endorsed by the publisher.
